# Three-Dimensional Mapping of mRNA Export through the Nuclear Pore Complex

**DOI:** 10.3390/genes5041032

**Published:** 2014-11-11

**Authors:** Steven J. Schnell, Jiong Ma, Weidong Yang

**Affiliations:** Department of Biology, Temple University, Philadelphia, PA 19122, USA; E-Mails: sj.schnell@temple.edu (S.J.S.); jiong.ma@temple.edu (J.M.)

**Keywords:** super-resolution microscopy, single-particle tracking, mRNA, nuclear export, nuclear pore complex

## Abstract

The locations of transcription and translation of mRNA in eukaryotic cells are spatially separated by the nuclear envelope (NE). Plenty of nuclear pore complexes (NPCs) embedded in the NE function as the major gateway for the export of transcribed mRNAs from the nucleus to the cytoplasm. Whereas the NPC, perhaps one of the largest protein complexes, provides a relatively large channel for macromolecules to selectively pass through it in inherently three-dimensional (3D) movements, this channel is nonetheless below the diffraction limit of conventional light microscopy. A full understanding of the mRNA export mechanism urgently requires real-time mapping of the 3D dynamics of mRNA in the NPC of live cells with innovative imaging techniques breaking the diffraction limit of conventional light microscopy. Recently, super-resolution fluorescence microscopy and single-particle tracking (SPT) techniques have been applied to the study of nuclear export of mRNA in live cells. In this review, we emphasize the necessity of 3D mapping techniques in the study of mRNA export, briefly summarize the feasibility of current 3D imaging approaches, and highlight the new features of mRNA nuclear export elucidated with a newly developed 3D imaging approach combining SPT-based super-resolution imaging and 2D-to-3D deconvolution algorithms.

## 1. Introduction

The nuclear pore complex (NPC), embedded in the nuclear envelope (NE), is a complex assembly of proteins that forms a gateway between the nucleus and the cytoplasm of eukaryotic cells. The number of NPCs in the nuclear envelope varies from hundreds (yeast) to thousands (human cells) [1,2,3,4,5,6,7]. As the major exchange pathway for proteins and genetic materials, the NPC (as a whole or its various subunits) is closely involved in the regulation and control of many cellular processes [8,9,10,11,12,13,14,15,16,17,18,19]. The structure of the NPC consists of an eightfold rotationally symmetrical core channel with an associated nuclear basket structure and eight cytoplasmic fibrils [10,13]. The NPC is approximately 60–120 MDa in mass, measures approximately 50 nm in diameter at the narrowest point (waist) and about 200 nm axially in total length in vertebrate cells [13,20,21,22] and represents the major portal to the nucleus by spanning the inner and outer nuclear membranes. Approximately 30 different proteins comprise the NPC structure, in multiples of eight copies each, for a total of approximately 400 proteins [20]. Approximately one third of these proteins are filamentous linker nucleoporins (Nups) rich in extensive phenylalanine-glycine (FG) repeats; these FG Nups are directly involved in regulating the selective transport of macromolecules through the NPC by forming a strict selective barrier, arguably in the form of a *polymer brush* or *hydrogel meshwork* [13,20,23,24,25,26]. In detail, passage of signal-independent small molecules (<40 kDa) across the NPC occurs by passive diffusion, whereas macromolecules with a nuclear localization signal or nuclear export signal require facilitated transport [21,27,28,29,30,31,32].

A large family of RNA molecules, messenger RNAs (mRNAs) play critical roles in the central dogma of molecular biology: transcription of DNA to RNA to protein [33]. After transcription and processing of primary transcript mRNAs by RNA polymerase, mature mRNAs transfer genetic information from DNA to the ribosome, where proteins are finally produced [34,35,36]. Specifically, in eukaryotic cells, mRNAs require export from the nucleus to the cytoplasm through the NPCs regulated by different signaling pathways [37,38]. Moreover, human mRNA-protein complexes (mRNPs), with sizes up to 100 MDa [39,40], generally require the transport receptors, such as NXF1-p15, to chaperone them through the NPC via interactions between transport receptors and the FG Nups [8,15,41,42,43,44,45]. Additionally, the human mRNP export process has been shown to be regulated by other protein complexes through differing mechanisms, including the transcription and export complexes (TREX and TREX-2) as well as factors involved in the release of cargo from the cytoplasmic surface of the NPC [46,47,48,49,50]. An additional mechanism has been discovered involving inositol polyphosphate multikinase (IPMK), which regulates transcript-selective nuclear mRNA export to retain genome integrity in humans [51,52]. The study of the mRNA export process and the details of the export path through the NPC are critical to the understanding of a vital step in the expression of genes in eukaryotes [14,53,54,55,56,57,58], as well as its potential uses in cancer immunotherapy, prophylactic vaccines, therapeutic gene products, or protein replacement therapies in the treatment and prevention of human diseases [59,60,61,62]. More precisely, locating the major selective barrier to nucleocytoplasmic export and a refinement of the measure of transport kinetics are important contributions to the understanding of the details of NPC’s role in mRNA export and other vital cellular processes, such as mislocalization of cytoplasmic proteins to the nucleus and deregulation of signaling pathways can have disastrous consequences (such as developmental defects or cancer) and directly or indirectly involve the interaction of the NPC with various proteins [63,64,65,66,67]. Additionally, it is believed that precise localization of molecular interactions within the NPC’s central channel itself is directly relevant to cancer-drug targeting strategies [68,69,70,71].

## 2. Single-Molecule Study of mRNA Nuclear Export in Live Cells

Conventional fluorescence microscopy can be used to image molecular dynamics in live cells, taking advantage of visualizing molecules of interest tagged with fluorescent proteins; however, the resolution of conventional light microscopy is generally considered to have a resolution limit of approximately half of the wavelength of the excitation light used, resulting in a resolution of approximately 250 nm in the x and y dimensions and about 750 nm in the z dimension [9,72,73,74,75,76,77,78] for commonly used visible excitation light. Given the size of the central channel of the NPC (~50 nm) and the passage of macromolecules with different sizes such as insulin (~4 nm), GFP (~6 nm) and mRNP complexes (up to 25 nm) [30,79], it is impossible to distinguish individual macromolecules within the NPC by conventional light microscopy. Whereas electron microscopy (EM) can achieve a level of resolution as high as <1 nm to observe single particles in the NPC [80] and cryo-EM using direct-detection cameras to enhance the recovery of high-resolution signal [81,82,83], preparation of the sample via chemical fixation or freezing prevents this technique from providing information about the real-time dynamics in live cells. To meet these challenges, single-molecule fluorescence microscopy techniques were developed to break the diffraction limit of conventional light microscopy by localizing the centroid of each fluorescent spot and tracking trajectories of biomolecules in real time with a spatial resolution of nanometer range under physiological conditions [84]. To date, several single-molecule techniques have been employed to characterize the movements of single mRNP particles through the NPC, finally bringing the dynamics of mRNA nuclear export into focus with one-dimensional (1D), two-dimensional (2D) and three-dimensional (3D) information [79,85,86].

### 2.1. 1D and 2D Single-Molecule Studies of mRNA Nuclear Export

With wide-field epi-fluorescence microscopy, Mor and colleagues tracked mRNPs containing large mRNA constructs with various sizes of 14 kb, 8 kb, and 4.8 kb in U2OS cells. The NPC and mRNA molecules were tagged with mCherry and with yellow fluorescent protein (YFP) fused to MS2 coat proteins, respectively. Multiple mRNP particles were visualized in the nuclear and cytoplasmic regions simultaneously; the authors concluded that the timeframe for transcription, transport and export of mRNA was estimated between 5–40 min in total. The temporal accuracy of their setup was 1 s per frame (dictated by the frame rate of the CCD camera) and allowed the authors to provide evidence for facilitated transport of mRNAs across the NE by demonstrating a transport rate 15 times faster than simple diffusion in nucleoplasm [79,80,81,82,83,84,85,86].

Soon after, to obtain the precision required to locate mRNA molecules relative to the nuclear envelope (NE), Grunwald and colleagues used super-registration epi-fluorescence microscopy to simultaneously image fluorescently labeled mRNPs and NE. In detail, the model they used was immortalized mouse cells engineered to express β-actin mRNA labeled with fluorescent YFP–MS2 tags and a nucleoporin (POM121) locating at the NPC center marked with tdTomato [79]. Then, emission fluorescence from YFP and tdTomato were separated by a dichroic filter and guided into two different cameras with an additional registration precision of ~10 nm [9,79,86]. With a 20-ms temporal resolution and a 26-nm spatial precision, they measured the mRNA export through the NPC consisting of a docking stage (80 ms), a transport step (5–20 ms), and a release process (80 ms), totaling 180 ± 10 ms in export time. The authors also provided 1D distribution of mRNP locations within the NPC and suggested a slow-fast-slow diffusion model for nuclear export of mRNPs through the NPC [79,86].

Differently, Siebrasse and colleagues used light-sheet microscopy for single-molecule detection of mRNA nuclear export [86]; in light-sheet microscopy the illuminating light takes the form of a sheet of optical plane that arrives at the sample perpendicular to the detection path. The laser is focused in one direction only with a cylindrical lens, producing a very thin sheet of light that allows for sectioning the sample optically [87]. Reduction of photobleaching, phototoxic effects, and high signal-to-noise ratio (SNR) comprise the advantages of light-sheet microscopy. The authors conducted their study in the *Chironomus tentans* system (salivary gland cells). To visualize native mRNPs during nuclear export, Siebrasse and colleagues labeled mRNPs by microinjecting an Alexa-fluor-647-hrp36 fusion protein, hrp36 being known to associate with mRNA during the mRNP assembly phase; the NE was labeled with Alexa-fluor-546-labeled transport receptor NTF2. The authors reported a spatial resolution of 10 nm and a temporal resolution of 20 ms; the transit time recorded ranged from 65 ms to several seconds, with that range attributed to the transport time being influenced by the varying sizes of the mRNPs being transported. A rate-limiting step was observed, which the authors surmised could be attributable to interactions at the nuclear basket of the NPC, and the success rate of mRNPs arriving in the cytoplasm was found to be approximately 25% [87].

**Figure 1:**
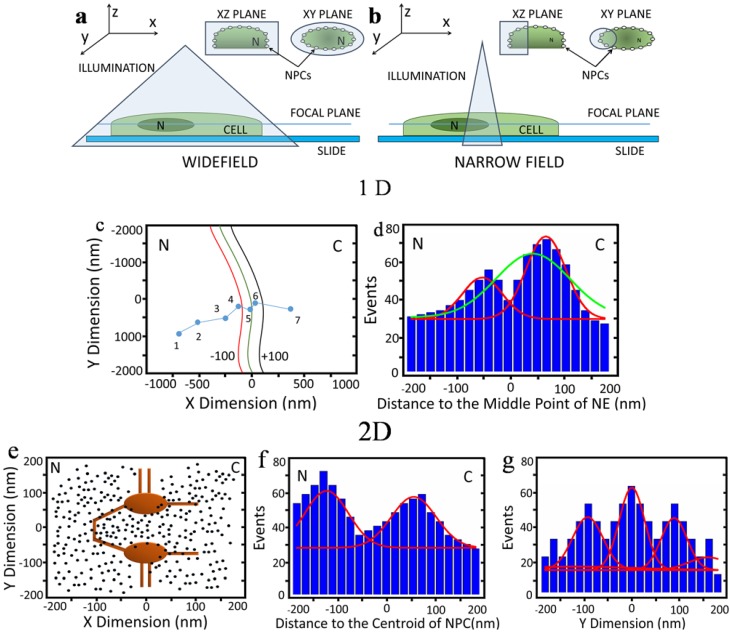
(**a**,**b**) Cartoon diagrams of illumination techniques of (a) wide-field and (b) narrow-field, showing volume of illuminated area. Note that in wide-field and narrow-field illumination, multiple nuclear pore complexes (NPCs) are illuminated; (**c**–**g**) Cartoon representations of data generated by various single-particle tracking (SPT) setups; (c) Typical 1D data showing path of an mRNA from the nucleus to the cytoplasm. The (green) center line is the experimentally determined position of the nuclear envelope (NE). The black (cytoplasm) and red (nuclear) lines are reference distances of 100 nm from the NE; (**d**) Histogram of the data in (c). Position of the molecule in 1D data is based on the position of the NE. Gaussian fitting could be interpreted as showing one (green) or two (red) areas of interaction of the molecule with the NPC; (**e**) An example of 2D data showing distribution of molecules (black dots) around the NPC (brown structure). N, nucleoplasm; C, cytoplasm; (**f**) Histogram of the distribution in (e) in the x dimension, showing fit of two Gaussian curves; (**g**) Histogram of the distribution in (e) in the y dimension, showing possible fit of multiple Gaussian curves. After Ma *et al.* [29] and Goryaynov *et al.* [9].

The above studies have provided great insight into the dynamic process of mRNA nuclear export by generating 1D and 2D information of single mRNP particles exiting the nucleus of live cells. The information obtained from the above measurements indicates single mRNPs exiting the nucleus by crossing the NE, rather than direct imaging of single mRNPs export through individual NPCs. Imaging single macromolecule transport through a specific NPC, however, represents a further step required to achieve 3D mapping of single-molecule trajectories in the NPC. For example, although the NPCs were marked with mCherry in the Mor study [85] and tdTomato in the Grunwald study [79], and Alexa546-labeled-NTF2 in the Siebrasse study [86], the final visualization of the location of NPCs was derived from the NE as a fluorescent line. The process by using the example of imaging the NE by wide-field and narrow-field epi-fluorescence microscopies is demonstrated in Figure 1. In detail, Figure 1 shows diagrams of wide-field (Figure 1a) and narrow-field (Figure 1b) microscopy setups and cartoon representations of the types of information that can be gleaned from these techniques. In either the wide-field or narrow-field imaging approach, multiple labeled NPCs on the NE are simultaneously excited to burst into fluorescence. Although NPCs are not uniformly distributed on the nuclear envelope (of HeLa cells in this example), the neighboring average distance of ~400–600 nm between NPCs [88,89,90] causes overlapped fluorescence of these excited NPCs, which prevents isolating individual NPCs but forms a clear fluorescent NE (Figure 1a,b). Normally, the middle line of the NE can be determined by line-scan of fluorescence intensity of the NE (Figure 1c). Detected in a different color, single-molecule trajectories of transiting molecules crossing the NE are later superimposed onto the fitted NE, providing evidence for further determining transport time and spatial distribution of the locations of these molecules (Figure 1d). Sometimes, researchers also arbitrarily overlaid single-molecule trajectories across the NE with an assumption that all these trajectories share a common central point, which would help produce a virtual 2D distribution of locations of single transiting molecules in the NPC, as shown in Figure 1e. Normally, standard samples (such as GFP molecules labeled with a red fluorophore [29]) should be used to align the system before trajectories are overlaid. Through this 2D distribution, information along each dimension could be further obtained (Figure 1f).

### 2.2. A Brief Summary of Currently Developed 3D Single-Particle Tracking (SPT) Techniques

**Figure 2:**
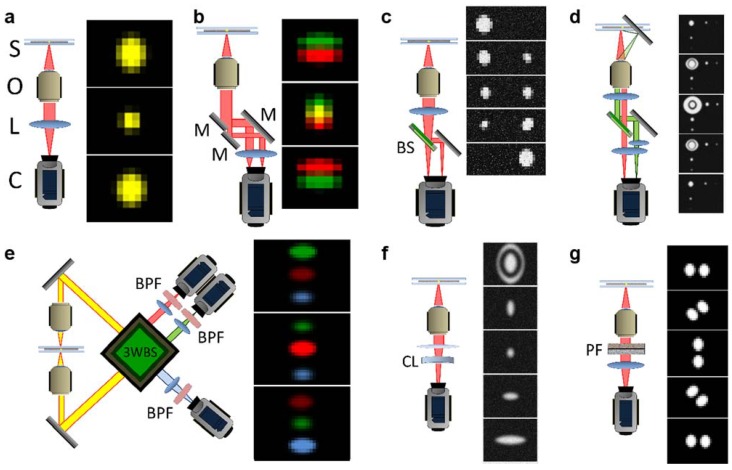
Diagrammatic representations of current major SPT setups. (**a**) Conventional fluorescence microscopy setup; (**b**) Parallax; (**c**) Biplane; (**d**) Angled mirror/side view; (**e**) Interference; (**f**) Astigmatism; (**g**) Double-helix point-spread function. S, sample; O, objective lens; L, lens; C, camera; M, mirror; BS, beam splitter; 3WBS, 3-way beam splitter; BPF, band-pass filter; CL, cylindrical lens; PF, phase filter. Distances between elements are not shown to scale and the positions of some elements are adjusted for clarity. After Deschout, *et al.* [91].

In the past years, numerous 3D single-particle tracking (3PT) techniques have been developed, as briefed in the following. But a concern exists whether these techniques could be readily applied to answer all of the questions posed in the study of nuclear transport. Figure 2 briefly shows diagrammatic representations of the major current 3D SPT setups.

Whereas conventional fluorescence microscopy is well described elsewhere [92,93,94,95], several modifications of the conventional setup (including the addition to the placement of colloidal microlenses in the immediate proximity of the sample [96]) have been made with the purpose of making 3D SPT possible. As shown in Figure 2b, parallax microscopy is achieved when the light beam is split into two nearly parallel light paths that together generate an image that consists of two images of the same particle; the z position is indicated when the two images exactly coincide; deviations from the particle’s actual z position result in the two images moving apart. An example of applying this technique is Sun and colleagues’ tracking of vesicles and single myosin molecules in living cells [97].

Similarly, in a multifocal technique, such as *biplane microscopy* [98], two or more focal planes are imaged simultaneously (Figure 2c), compared to a single focal plane view in conventional light microscopy (Figure 2a). Using *biplane microscopy* as an example*,* the addition of a second focal plane is accomplished by inserting a beam splitter in the path of emitted fluorescence from the sample so that two images formed at different focal planes are now directed to two different cameras or two regions of a same camera. Comparison of the positions of the imaged particle in the two different focal planes provides information about the movements of the particle in z direction. This technique was used, for example, by Prabhat and colleagues to demonstrate tracking of the movement of particles along a tubule along the part of the cellular recycling pathway that spans from the sorting endosome to exocytosis at the plasma membrane [99]; this technique was used also by Toprak and colleagues to track the movements of melanosomes and phagocytosed fluorescent beads [100].

As shown in Figure 2d, *angled mirror/side view* microscopy is a technique in which the sample is mounted on a substrate that incorporates micro-mirrors that provide orthogonal reflections of the fluorescent particle. The reflections of the particle move laterally with z-dimensional movement of the particle, which can be combined with the lateral movements of the particle image proper to reconstruct information about the movement of the particle in three dimensions. This technique was used by McMahon and colleagues to track fluorescent-dyed polystyrene spheres in an aqueous medium over the mirror-containing substrate [101] and by Tang and colleagues to image protein distribution in bacteria [102].

Caused by different levels of interference between light beams, *interference* microscopy [78] is a technique in which the emitted light beam is split multiple times and the beams allowed to interfere with one another in such a way as that differences in z position are represented by differing light intensities (Figure 2e). This technique was used by Aquino and colleagues to image the distribution of fibrinogen receptors on human platelets and tubules in mammalian cells [103] and by Shtengel and colleagues to image various cellular structures, including microtubules [104].

To change the shape of the illumination point-spread function (iPSF) for fluorescent particles, *astigmatism* microscopy involves the use of a cylindrical lens to introduce an astigmatic aberration into the light path (Figure 2f); whereas a particle in the focal plane is the more usual round shape, deviation from the focal plane is made evident by an increasing ellipsoidal appearance of the particle, providing information about its z position. This technique was used by Kao and colleagues to track fluorescent particles in aqueous solution and in living cells [105] and by Huang and colleagues [106] to image polystyrene beads in aqueous solution and clathrin-coated pits in cells.

In *double helix PSF* (Fourier) microscopy (Figure 2g), the excitation PSF of the microscope is designed such that it is comprised of two lobes, the angle of the line between which varies with the z position of the imaged molecule, resulting in a double-helix shaped PSF oriented along the z axis [107,108]. Pavani and colleagues used this technique to image fluorescent beads [109] and Thompson and colleagues used it to identify some apparently nonrandom motions of mRNA in live yeast cells [107].

Basically, *biplane*, *angled mirror*, and *astigmatism* microscopies all use a PSF to fit against z position. However, moving particles provide an average position; when convoluted with diffraction, the PSF is less accurate, because measured data will instead correspond to the convolution of the PSF [108] with the spread of the molecule along its path. In the other techniques, the emission fluorescence from single particles is divided into different channels; each channel possesses weaker signal with a lower SNR and a low spatiotemporal resolution. These techniques can be potentially utilized to track single mRNPs export through the NPC in live cells, but they would need further modifications to acquire the spatiotemporal resolution to answer questions about transient transport kinetics and 3D specific paths within the sub-micrometer-sized NPC.

### 2.3. 3D Mapping of mRNA Nuclear Export with SPEED Microscopy

**Figure 3:**
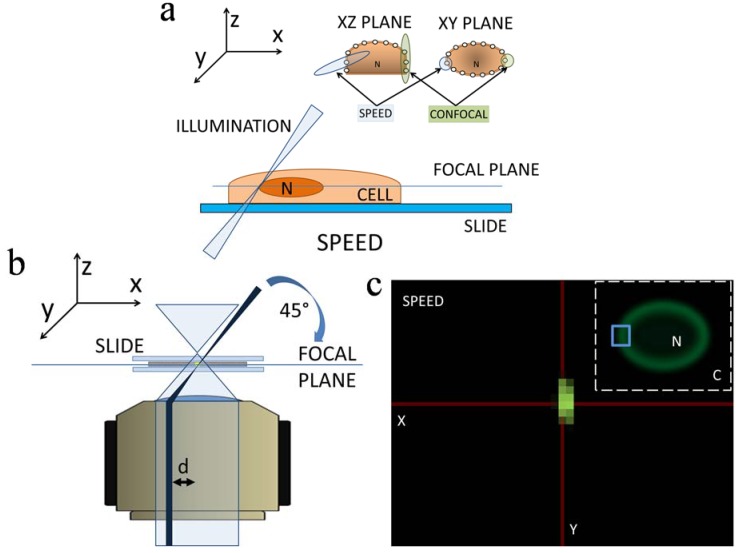
(**a**) Cartoon diagram of the illumination techniques of SPEED microscopy, showing volume of illuminated area (light blue) compared with that of confocal microscopy (light green). Note that only one NPC is illuminated with SPEED; (**b**) Cartoon diagram of the optics of the SPEED setup, showing the excitation beam paths of the SPEED (dark blue) and wide-field epifluorescence (light blue). The illuminating laser beam in the SPEED setup is focused into a diffraction-limited spot in the focal plane from the edge of the objective at a 45° angle to the focal plane; (**c**) Cartoon diagram showing multiple labeled NPCs excited using wide-field epifluorescence; the area under study encompasses one NPC, upon which the blue box is centered (inset). After Goryaynov *et al.* [9] and Ma *et al.* [29].

To further develop 3D SPT techniques and bring into focus details of mRNA nuclear export that have escaped previous 1D and 2D studies, our laboratory has employed s*ingle-point edge-excitation sub-diffraction* (SPEED) microscopy to conduct SPT of single mRNP translocation through the NPC of live HeLa cells with a spatiotemporal resolution of 8 nm and 2 ms [29,30], in a manner of illuminating single mRNPs through single NPCs and mapping 3D export route for mRNPs, as shown in Figure 3. Technical advances in SPEED microscopy are attributed to the following features in its setup: (1) most current single-molecule studies of nuclear transport are typically conducted along the equatorial plane of the generally disk-shaped nuclei of HeLa and other cells, aiming at easily determining the moving direction of targeted substrates through the NPC between cytoplasm and nucleus. However, a perpendicular illumination volume, like that of the stationary mode of confocal microscopy, cannot avoid illuminating several GFP-NPCs with overlapped fluorescence on the NE, particularly in the z dimension. In our setup, as shown in Figure 3a,b, an inclined illumination PSF (~320 nm in x, y, and z dimensions), smaller than the average nearest neighboring distance between the NPCs on the NE, enabled the excitation of a single GFP-labeled NPC only in each detection (Figure 3c). Additionally, because NPCs are not homogenously distributed in HeLa cells (approximately 3–6 pores/µm^2^ [7], we choose the least densely populated region of the NE for study to enhance imaging a single NPC in the illumination volume of SPEED microscopy; (2) The high optical density (100–500 kW/cm^2^) of the small iPSF also squeezes out a high number of photons in a short time period from a single mRNP particle tagged with approximately ten copies of mCherry [30]. Typically, more than five thousand photons are obtained from a single mRNP molecule within a 2-ms detection time. To reduce any photobleaching and phototoxic effects, an optical chopper (Newport, Franklin, MA, USA) is used to create an on/off operational mode with off-time ten-fold longer than on-time [29]; (3) The inclined iPSF further greatly avoids out-of-focus background fluorescence and auto-fluorescence of the objective [29,30], which enhances a SNR higher than 11 and a spatial resolution better than 10 nm; (4) The small illumination volume of SPEED microscopy allows the imaging of single molecules within a small pixel area of the CCD camera, resulting in a very fast detection speed (up to 5000 frames per second if needed).

Supported by these new features, the SPEED methodology was further developed in two aspects: Firstly, 2D SPT data within single NPCs were acquired by tracking single mRNPs through a singly illuminated NPC with a spatiotemporal resolution of 8 nm and 2 ms; secondly, the inherent 3D pathways of the mRNPs in the NPC were recovered via a 2D-to-3D deconvolution algorithm by utilizing the structural rotational symmetry of the NPC and the superposition of thousands of single-particle trajectories collected from multiple NPCs [6,9,30,110]. The uniform rotational distribution of single molecules at the cross-section of the NPC, an assumption made in our deconvolution process, is based on the fact of eightfold radial symmetry of the pore as elucidated in EM studies [6], enhanced by lack of the referent points at θ-dimension in the cylindrical coordinate system as multiple NPCs are overlaid, and finally confirmed by the experimental single-molecule measurement of spatial distribution of single transiting molecules at the cross-section of NPC locating at the bottom of the NE (Supporting Information of Ref. [29]).

**Figure 4:**
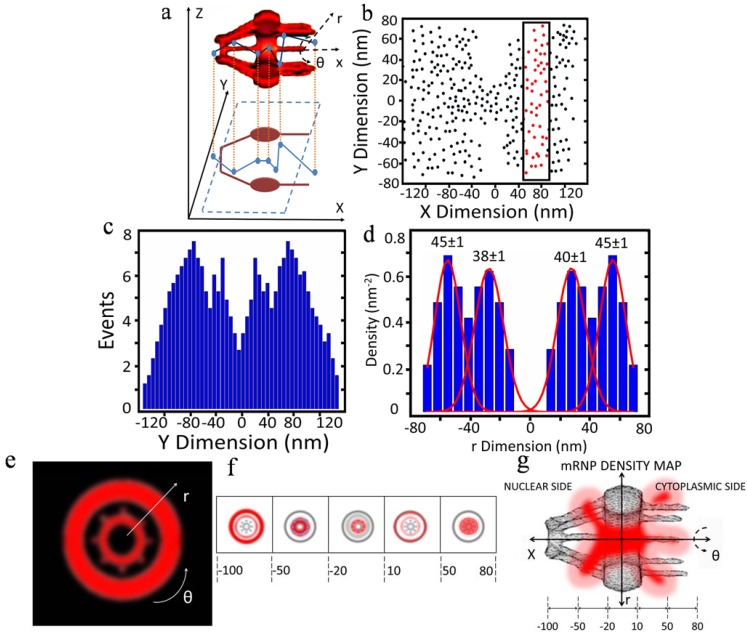
Cartoon representations of 3D data generated with SPEED microscopy. (**a**) Plot of the interaction sites of a molecule (mRNP in this example) in the NPC, showing 3D spatial locations of a single tracked molecule projected (*x*, *y*, *z*) onto a 2D representation of the NPC. The 3D spatial locations are described via cylindrical coordinate system (*r*, θ, *x*). Coordinate x represents the location along the NPC axis, whereas (*r,* θ) refers to the positions at the cross-section of the NPC; (**b**) 2D plot of events from many molecules tracked. The x and y dimensions are shown in nanometers. The 2D spatial locations of events within the NPC are spatially divided into sections proceeding along the axial direction. One section is highlighted and will be further analyzed in this example; (**c**) Histogram of the section of the plot highlighted in (b), showing spatial locations of events in the y dimension; (**d**) Corresponding histogram in the r dimension, obtained by deconvolution process, showing major peaks shown by Gaussian fitting; (**e**) Radial distribution (cross-sectional image) of the region in (d); (**f)** Radial distributions are assembled from all sections of the pore (demarcated relative axial distances from the centroid of the NPC shown in nm), to create (**g**) The five cross-sectional regions shown in (f) are assembled to generate a spatial density map (cutaway view shown; *r*, *θ*, *x* dimensions; demarcated relative axial distances from the centroid of the NPC shown in nm). After Ma *et al.* [29] and Goryaynov *et al.* [9].

Cartoon representations of data output typical of the SPEED system are shown in Figure 4. The obtained 2D locations are a projection effect of the actual 3D locations as indicated in cylindrical coordinates (Figure 4a) [29]. In detail, firstly thousands of single-molecule transport events through the NPC are superimposed in two dimensions (Figure 4b). Then, a histogram of spatial locations is generated from an axial slice or region of the NPC (squared red dots in Figure 4b). Next, the histogram is used to deconvoluted (as well as called back-projected) to a radial distribution of densities in that region of the pore (Figure 4c,d). Finally, by combining all the concentric rings plotted from the radial distributions from each region of the pore, a complete 3D spatial density map of mRNA molecules within the NPC was finally generated (Figure 4e–g).

## 3. New Features of Nuclear Export of mRNA Obtained with 3D Mapping

By 3D mapping of real-time nuclear export of mRNPs through the NPC with SPEED microscopy, our laboratory revealed several important new features that escaped previous studies. First, the location of major selective barriers for mRNPs was identified inside the NPC. We found that approximately 36% of all exporting events of mRNPs successfully complete their export journey, arriving in the cytoplasm after they conquered the barrier locating in the nuclear basket and the central scaffold region of the NPC, whereas the other 64% of mRNPs failed their export mainly before they passed the central scaffold region. Very likely, FG Nups located at the central scaffold region of the NPC would play the dominant role in gating which mRNP molecules should continue their transport into the cytoplasm. However, the exact causes for the abortive nuclear export events of mRNA are not completely understood yet. Current accumulation of evidence suggest several possible reasons including the remodeling and preparation of mRNP for transport by cofactors and adaptor proteins such as TREX, TREX-2 and GANP at the nucleoplasmic face of the NPC, the physical constrain for mRNP particles at the narrowest scaffold region of the NPC and the dissociation process involving Dbp5 and Gle1 on the cytoplasmic side of the NPC [30,32,34,111,112,113]. Second, a refined nuclear export time of ~12 ms for mRNPs was obtained. The newly determined export time of mRNPs is much shorter than previously reported. The difference is at least partially attributed to the faster detection speed and the high localization precision of localizing an individual NPC. For comparison, we repeated the measurements with a slower detection speed, resulting in a longer nuclear export time for mRNPs. Also, we found there are many slowly moving mRNPs at the periphery of NE, which could be mistakenly included in counting nuclear export time for mRNPs if single NPCs could not be precisely localized. Third, a new diffusion pattern of mRNPs in the NPC was revealed by this high-resolution 3D imaging approach. The diffusion coefficient of mRNPs in each sub-region of the NPC was directly determined as: ~0.16 μm^2^/s on the nucleoplasmic side, ~0.07 μm^2^/s in the central scaffold region, and ~0.19 μm^2^/s on the cytoplasmic side, revealing a fast-slow-fast diffusion pattern adopted by mRNPs to export through the NPC. Fourth, a 3D reconstruction of the export route revealed the actual pathway of mRNPs through the NPC in live cells for the first time. More interestingly, mRNPs, the large RNA:protein complexes, primarily interact with the periphery of, and seldom present in, the central axial channel that is reserved for small molecules to passively diffuse through on the nucleoplasmic side and in the center of the NPC. Fifth, the 3D path indicated that mRNPs dissociate on the cytoplasmic side of NPC, at which point mRNPs have a low probability of returning to the nucleoplasm and start extending into the previously unoccupied axial channel.

## 4. Conclusions

With the great advances in single-molecule microscopy imaging techniques, the detailed dynamics of mRNP nuclear export have been finally brought into focus. Particularly, 3D super-resolution mapping approaches reveal many new features that escaped previous 1D and 2D single-molecule studies of mRNA nuclear export, such as 3D export route, location of selective barrier, refined export time, and diffusion modes within the sub-micrometer-sized native NPC. In the near future, we expect to expand current high-resolution 3D imaging methodologies to investigate nucleocytoplamic transport mechanisms of other types of RNAs, such as rRNA, microRNA and viral RNA. Additionally, we also plan to employ the technique mapping 3D tomography of macromolecule trafficking in other sub-micrometer bio-cavities or organelles.
